# Women's Estrus and Extended Sexuality: Reflections on Empirical Patterns and Fundamental Theoretical Issues

**DOI:** 10.3389/fpsyg.2022.900737

**Published:** 2022-06-20

**Authors:** Steven W. Gangestad, Tran Dinh

**Affiliations:** Department of Psychology, University of New Mexico, Albuquerque, NM, United States

**Keywords:** menstrual cycle, fertility, sexual desire, mating, ovulation

## Abstract

How do women's sexual interests change across their ovulatory cycles? This question is one of the most enduring within the human evolutionary behavioral sciences. Yet definitive, agreed-upon answers remain elusive. One empirical pattern appears to be robust: Women experience greater levels of sexual desire and interest when conceptive during their cycles. But this pattern is not straightforward or self-explanatory. We lay out multiple possible, broad explanations for it. Based on selectionist reasoning, we argue that the conditions that give rise to sexual interests during conceptive and non-conceptive phases are likely to differ. Because conceptive and non-conceptive sex have distinct functions, the sexual interests during conceptive and non-conceptive phases are likely to have different strategic ends. We discuss provisional evidence consistent with this perspective. But the exact nature of women's dual sexuality, if it exists, remains unclear. Additional empirical research is needed. But perhaps more crucially, this topic demands additional theory that fruitfully guides and interprets future empirical research.

## Introduction

In *The descent of man and selection in relation to sex*, Darwin (1871) introduced the concept of *sexual selection*: “We are, however, here concerned only with that kind of selection, which I have called sexual selection. This depends on the advantage which certain individuals have over other individuals of the same sex and species, in exclusive relation to reproduction” [p. 256). Sexual selection received scant attention for a century following this debut (an important exception being Fisher (1930)]. A volume dedicated to the 100-year anniversary of Darwin's book, edited by Campbell (1972), prompted a sea change. In one chapter, Mayr (1972) tackled the important question of what exactly discriminates sexual selection from natural selection, an issue still debated today. In a book review, Williams (1973) offered the prescient observation that Trivers's (1972) chapter on parental investment and sexual selection was among “what may be the most permanently valuable part of the book” (p. 788)—in retrospect, a vast understatement. Its legacy owes more to it promoting adaptationist analysis in *strategic* terms than to particular claims about sexual selection (some of which have been revisited; e.g., Kokko and Jennions, 2008). As Williams summarized, “An organism is represented, in effect, as a player in a game the object of which is to maximize the representation of one's genes … in the population to which one belongs. Sexual reproduction and family life are seen as a complex system of mutual exploitation, conflict, compromise, and cautious coalitions, with each player totally committed to maximizing its own score” (Williams, 1973, p. 788).

As evolutionary psychology sprouted from adaptationist frameworks inspired by Williams, Trivers, Hamilton, and a host of their contemporaries (e.g., Tooby and Cosmides, 1992), it is no wonder that its *functional* analyses of psychological adaptations are very often *strategic* analyses; that is, the functions of psychological adaptations are often understood in terms of how they facilitate individuals' strategic aims.

In this paper, we discuss a long-standing issue in evolutionary behavioral science: How women's sexual interests shift across their menstrual cycles from conceptive to non-conceptive phases, purportedly largely through regulatory effects of ovarian hormones. Conceptive and non-conceptive sexual phases very likely have distinct functions and, hence, ancestrally benefitted women in different ways. A functional analysis therefore leads to a strategic analysis. How do women's sexual interests during different phases of the cycle strategically promote their fitness interests? Though proposals to date have identified some strategic shifts (e.g., increased sexual interests when women are conceptive may “strategically” lead to increased rates of conception), additional strategic analyses (perhaps especially of non-conceptive sexuality) are needed. Our paper is a conceptual one; we do not offer strong claims with respect to empirical patterns. We argue that progress toward understanding human sexual selection and the evolution of human mating could benefit from a more thorough-going commitment to strategic analysis in this domain.

## Women's Purported Loss of Estrus

*Estrus is the relatively brief period of proceptivity, receptivity, and attractivity in female mammals that usually, but not invariably, coincides with their brief period of fertility. Human females do not experience estrus. … [E]strus must have been lost at some point in human ancestry*. (Symons, 1979, p. 97).

*Beach goes on to say, “Although human females are not continuously ‘sexually receptive,’ they are continuously ‘copulable’; and their sexual arousability does not depend on ovarian hormones. This relaxation of endocrine control contributes to the occurrence of coitus at any stage of the menstrual cycle” (pp. 357–358). I believe that this is the clearest available statement of what the “loss of estrus” means*. (Symons, 1979, p. 106).

How do women's sexual interests change across their ovulatory cycles? This question is one of the most enduring within the human evolutionary behavioral sciences. Psychological changes across women's cycles have long been thought to embody design features important to inferring the nature of selection pressures that uniquely shaped human sociality. In the two quotes above, from *The Evolution of Human Sexuality*, Symons (1979) states, first, that estrus was lost in women (see also Lancaster and Lee, 1965; Jolly, 1972) and, second, that this loss of estrus effectively amounts to a relaxation of endocrine control over women's sexual arousability. In Symons' view, a key to understanding the evolution of human sexual relations is explaining why women lost estrus and became capable of experiencing sexual arousal across the cycle. Within just a few years following publication of his book, a number of accounts were proposed (Alexander and Noonan, 1979; Benshoof and Thornhill, 1979; Burley, 1979; Symons, 1979).

Nearly four decades later, scores of studies have sought to investigate how women's sexual desire, sexual interests, mating priorities, and mating behavior systematically change across the cycle, as well as the hormonal contributions to these changes (for partial reviews, see Thornhill and Gangestad, 2008; Roney and Simmons, 2013; Gildersleeve et al., 2014a; Gangestad et al., 2021; Roney, 2021; Stern and Penke, 2021; Havlíček and Roberts, in press). A tremendous amount of progress has been made. For instance, the robustness of some empirical patterns that was once questioned is now well-established. On average across women, robust mid-cycle increases in sexual desire has been repeatedly demonstrated (reviewed below in the section, “Change in Sexual Interests Across the Cycle”). Yet there is no clear agreement about these how changes (or lack thereof) should be understood. A variety of theoretical perspectives, which predict different empirical patterns, have been proposed. None is near-universally accepted. Despite some robust empirical patterns, the literature is marked with several large-scale failures to replicate effects once thought to be well-established. Hence, ambiguities about what basic phenomena exist and require explanation persist, a major reason why fundamental questions endure.

This paper reflects on this literature and the key theoretical issues that persist. It consists of four major sections.

First, we begin by discussing some generally well-established empirical patterns. In particular, studies generally support the notion that ovarian hormones affect mean levels of sexual interest. During cycle phases with elevated estradiol and/or diminished progesterone levels, women experience, on average, greater levels of sexual interest.

Second, we describe two broad, alternative perspectives on the nature of female sexual interests and hormonal effects on sexual interests. One perspective argues that hormones affect libido—a generalized state of increased sexual interest. A second perspective views sexual desire as evoked by specific circumstances (sexual “incentives”). In this view, hormones affect the circumstances that evoke sexual interest—that is, hormones moderate the influence that particular conditions and mate features have on sexual interests.

Third, we discuss the evolution of “extended sexuality”—female sexuality during phases when sex cannot lead to conception. Naturally, non-conceptive sex evolved to serve functions other than conception. We illustrate this point with instances of extended sexuality in non-human primates. We argue that, given that extended sexuality *functions* differently from conceptive sexuality, the adaptive *strategies* embodied within extended sexuality should differ from those embodied within conceptive sexuality. Hence, on grounds of *a priori* evolution-inspired theory, the circumstances that evoke sexual interests during extended sexual interests during non-conceptive phases should differ from those that evoke sexual interests when sex is conceptive.

Fourth, we briefly discuss literature in light of these expectations. We describe several patterns consistent with the idea that the conditions that evoke sexual interests when women are conceptive do not perfectly match the conditions that evoke sexual interests when they are non-conceptive. Nonetheless, much more empirical work is needed in this domain. Indeed, theoretical avenues necessary to make sense of phenomena in this domain have yet to be fully laid out and explored. We also note theoretical implications of the possibility that expected patterns are not realized.

## Change in Sexual Interests Across the Cycle

In a remarkable study, Roney and Simmons (2013) asked 43 naturally ovulating women to complete a daily diary, which included daily ratings of women's sexual desire, over the course of up to two full cycles. Saliva was collected nearly every day for assays of estradiol, progesterone, and testosterone. In a mixed model regression analysis, within-woman variations in estradiol levels (characteristically high during the conceptive, late follicular phase) positively predicted sexual desire, whereas within-woman variations in progesterone levels (characteristically low during the late follicular phase) negatively predicted it. These associations gave rise to a peri-ovulatory rise in sexual desire.

Other studies have also yielded evidence that women experience heightened sexual interest during the peri-ovulatory phase. In a study of 35 women followed across a cycle, Mass et al. (2009) similarly reported a follicular phase peak in self-reported sexual desire. Women were also video-recorded while viewing a series of erotic pictures of attractive, masculine men and non-sexual control stimuli (kittens and rabbits). During the follicular phase, women exhibited more expressions associated with pleasure when viewing nude men. Rudski et al. (2011) asked women to describe implicitly erotic art (e.g., Georgia O'Keefe's flower paintings). Descriptions written in the follicular phase contained markedly more sexual references than those written during the luteal phase. Near ovulation, women's dreams include greater sexual content (Natale et al., 2003) and, when given a choice of a film to view, they are more likely to choose an erotic one (Zillmann et al., 1994). From a study of 259 naturally cycling women followed over two cycles, Prasad et al. (2014) reported a mid-cycle spike in sexual activity linked with higher same-day estradiol and LH levels (though they did not detect an impact of progesterone levels). Another study that followed several hundred women over five sessions found negative impacts of progesterone on sexual desire (Jones et al., 2018a). A number of studies have reported midcycle increases in women's attraction to men other than primary partners (Gangestad et al., 2002, 2005; Durante and Li, 2009; cf. Jones et al., 2018a; Shirazi et al., 2019b). In large samples of partnered women followed across at least one full cycle, Arslan et al. (2018) found robust increases in both in-pair and extra-pair sexual interest with increased conception risk (see also Shirazi et al., 2019a). And, in a study of ~3 million women from nearly 150 countries worldwide, Pierson et al. (2021) reported that women were ~3–5% more likely to engage in sex during the peri-ovulatory phase as compared to the mid-luteal phase. Though some studies have failed to detect similar associations (e.g., Slob et al., 1991; Meuwissen and Over, 1992; Suschinsky et al., 2014), the total body of evidence seems very clear: *On average, women experience greater levels of sexual interest during the late follicular/peri-ovulatory phase than during other phases of the cycle* (especially when compared to the luteal phase; see also Krug et al., 1994, 2000; Van Goozen et al., 1997; Bullivant et al., 2004; Wilcox et al., 2004)^1^.

## Interpreting Variation in Sexual Desire Across the Cycle: Libido vs. Incentive Perspectives

How do these findings speak to the forms of ancestral selection that shaped women's sexual interests? To our minds, these empirical patterns are not straightforward or self-explanatory. We should ask, what *explains* the observation that, on average, women experience greater levels of sexual interest during the peri-ovulatory phase than during other phases of the cycle?

### The Nature and Function of Female Sexual Desire

The motivational priorities perspective seeks to explain shifts in women's sexual desire, based on the argument that the potencies of women's motivations change across the cycle (see Roney and Simmons, 2013, 2016, 2017; Roney, 2018, 2021). When women can potentially conceive, their mating motivations (e.g., sexual interests) assume greater priority. Other motivations (e.g., motivation to eat; Roney and Simmons, 2017) assume less priority. Relative priorities shift during cycle phases when women cannot conceive. Put simply, women should be most motivated to have sex when it has the greatest fitness benefits, and it has the greatest fitness benefits when it can result in conception (For additional discussions of this view, see Jones et al., 2018b; Jünger et al., 2018a).

In contrast to these views, the dual sexuality framework conceptualizes women's estrous and extended sexuality as taking distinct forms—with partly distinct functions arising from different costs and benefits of sex during conceptive versus non-conceptive phases (Thornhill and Gangestad, 2008; for an in-depth discussion of the dual sexuality framework, as well as differential costs and benefits of sex during conceptive and non-conceptive phases, see Gangestad et al., 2021). The circumstances that evoke estrous and extended sexual interests are expected to differ. Therefore, specific conditions and stimuli likely moderate hormone-associated changes in women's sexual interests. This can produce the small, positive observed associations between conception probability and sexual desire, *on average across women*. We return to further discussion of dual sexuality below.

#### Sexual-Desire-as-Libido

What does it mean for *sexual desire* to vary across the cycle? Roney and Simmons (2013) introduce their study as one that examines “physiological signals that regulate cyclic patterns of libido” (p. 636). The concept of libido was introduced by Freud (1953): “The fact of the existence of sexual needs in human beings and animals is expressed in biology by the assumption of a ‘sexual instinct,’ on the analogy of the instinct of nutrition, that is of hunger. Everyday language possesses no counterpart to the word ‘hunger,’ but science makes use of the word ‘libido’ for that purpose” (p. 135). Setting aside Freud's psychoanalytic explication of libido, the fundamental premise is that libido is an *internally generated* energy—a “drive”—that motivates sexual activity, and that is satiated by achieving sex. In much the same way that hunger motivates and is satiated by food consumption, the “source [of libido] is a state of excitation in the body, its aim is the removal of that excitation; on its path from its source to its aim the instinct becomes operative psychically. We picture it as a certain quota of energy which presses in a particular direction. It is from this pressing that it derives its name of 'Trieb' (literally ‘drive’)” (Freud, 1964, p. 96).

#### An Alternative Perspective on Sexual Desire

An incentive motivational framework offers an alternative conceptualization of sexual motivation (e.g., Both et al., 2007; Toates, 2009). In this perspective, motivational states are *not* internally generated. Rather, organisms are motivated to act in particular ways when presented with *incentive*—a structured environment that promises rewarding outcomes potentially achievable through particular behaviors, which are then motivated. Drive versus incentive perspectives are often distinguished in terms of “push” versus “pull” metaphors: Whereas drive perspectives view motivation as forces that “push” an individual to engage in particular behaviors, incentive perspectives view goal-directed behavior in terms of “pulls” from the environment, which can also be thought of as attractions. Sexual-desire-as-libido arises prior to and independent of *in situ* attraction. By contrast, the incentive motivational framework argues that sexual desire cannot possibly be distinguished from sexual attraction; attraction and desire are inextricably related^2^.

That said, internal states, independent of attraction, play highly important roles within an incentive perspective. Rather than directly affect motivation *per se*, however, they *potentiate* (or *de-potentiate*) incentives or attractions—that is, render them more or less potent elicitors of sexual interest. Hence, internal states modulate the activation of motivation, contingent on eliciting conditions. A persistent reduction of sexual desire, then, was not a loss of internal “drive” (libido). Rather, the lack of sexual desire is a result of conditions that do not incentivize experiences of sexual attraction—i.e., an individual's sexual response system was not activated by the circumstances the individual encountered (though other circumstances that were not encountered may or may not prompt sexual responses)^3^.

A variety of factors can modulate the potentiation of incentives. In many species, hormones play crucial roles (See Michael, 1993, for an explicit discussion of female hormonal changes as “establishing operations,” events that modulate potentiation of reinforcing consequences). In mammalian species in which females exhibit classic estrus—they are sexually receptive or proceptive *only* during a discrete period coinciding with their capacity to conceive—female sexual incentives are potentiated *only* during specific hormonal states. (These often involve multiple hormones, but most consistently the family of estrogens [named, literally, for being the “generators of estrus”; Allen and Doisy, 1923]). Even when female capacity to be sexually attracted to and interested in males is not fully dependent on ovarian hormones, ovarian hormone levels can upregulate or downregulate the sensitivity of females' sexual response system to yield attraction and, hence, desire.

#### Implications for Understanding the Function of Sexual Desire

A fundamental difference between perspectives on libido and incentive involves how internal states modulating the experience of sexual desire are conceptualized. If sexual desire can be understood as libido, internal states drive sexual motivation, independent of external stimuli. From an incentive perspective, these internal states alter the *potential* for sexual interest; still, sexual interest is contingent on encountering a sexually attractive stimulus, the attractiveness of which depending on the internal state. But what are the implications for viewing sexual desire as solely internally-driven or as responsive to external stimuli?

In fact, implications for understanding the evolved functions of sexual desire run deep. Let us consider a species that exhibits classic estrus—females have evolved to be sexually motivated to mate with male conspecifics only during the estrous phase. The perspective that views sexual desire as internally driven, rather that stimulus-evoked, may imply that the primary function of sexual desire is to obtain sperm (i.e., to conceive). As Fisher (1998) asserted, “The sex drive (the libido, or lust) is characterized by the craving for sexual gratification; […] it evolved primarily to motivate individuals to seek sexual union with *any* conspecific” (Fisher, 1998, p. 24; cited in Toates, 2009). In his 1979 book, Symons argued that this perspective makes very little sense from modern evolutionary biology. Alternative males differ with respect to genotypic and phenotypic features, which then render them better or worse sires. *Which* male sires a female's offspring has fitness consequences for the female. Hence, selection should shape mechanisms to bias sire choice toward males offering relatively high fitness prospects and away from males offering low fitness prospects. From an incentive perspective, sexual attraction therefore partly functions to affect sire choice (during estrus; below, we discuss other potential functions outside of estrus). Accordingly, discriminatory sexual attraction constitutes a much better *strategy* than does indiscriminant attraction and desire (see also Thornhill and Gangestad, 2008)^4^.

### Interpreting Variations in Sexual Interests

We now return to the question we posed earlier: What does it mean for women's sexual desire to vary across the cycle? Depending on whether we conceive of sexual desire as libidinous drive or contingent on eliciting conditions, differences in levels of desire across the cycle could arise in a variety of distinct ways.

For one, hormones could affect libido and, hence, affect an internal state of sexual desire, independent of eliciting circumstances (e.g., Roney, 2018, 2021).

Alternatively, hormones could affect the “incentives” that evoke sexual interests—that is, the potency with which circumstances evoke sexual interests, the potency with which male features evoke sexual interests, and/or higher-order contingencies that involve both circumstances and male features. Under particular hormonal conditions, one circumstance may heighten the potency of some male features, whereas another circumstance may heighten the potency of other male features. Under other hormonal conditions, these same contingencies may not exist.

**Figure 1:**
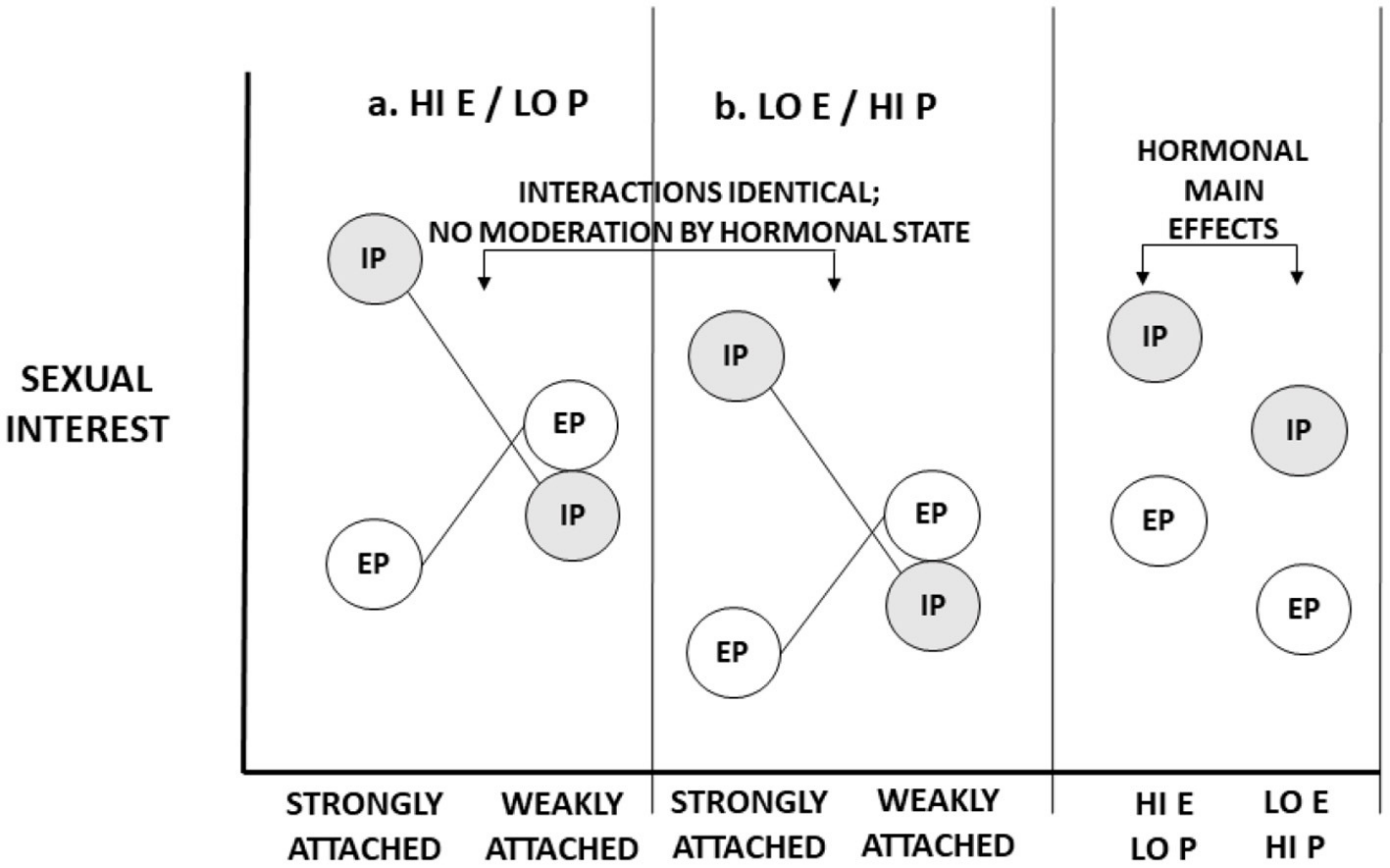
Illustrative representation of the impact of ovarian hormones on “libido”—generalized sexual desire. Two hormonal states are represented: **(a)** High levels of estrogen (E) and/or low levels of progesterone (P), characteristic of the conceptive, peri-ovulatory phase; **(b)** (relatively) low levels of estrogen and high levels of progesterone, characteristic of the non-conceptive, luteal phase of the cycle. Two conditions are represented: Women in relationships in which they are strongly attached and bonded to romantic partners; and women in relationships in which they are weakly attached and bonded with partners. Sexual interests in partners (in-pair [IP] sexual interests) and men other than partners (extra-pair [EP]) sexual interests are considered. Hormonal states have main effects on sexual interests. Values in panel A “add” a constant value to sexual interest. This constant value is reflected in an overall main effect on both IP and EP sexual interests.

We partially flesh out these separate possibilities with two figures. In Figure 1, we illustrate the impact of purported libido on sexual interest. For purposes of the illustration, we consider just two circumstances, both concerning a partnered woman. In one circumstance, she is strongly attached to her partner. In the other circumstance, she is not strongly attached to her partner. We consider one male quality: whether the male is her partner (in-pair) or a non-partner (extra-pair). We represent two hormonal states—the state of high estradiol and/or low progesterone (a conceptive hormonal state); and the state of low estradiol and/or high progesterone (a non-conceptive hormonal state). If hormonal states affect libido, then hormonal states affect sexual interest in all men across all circumstances. Naturally, circumstances may affect sexual interests, male partnership status may affect sexual interests, and circumstances may interact with male features to affect sexual interests (e.g., when women are strongly attached to partners, they may be especially sexually interested in partners as opposed to non-partners). But *hormones* do not *interact with* circumstances or male features to affect interest; hormonal effects on libido are solely main effects, independent of other factors (“inputs”) that affect sexual desire (see Roney, 2018, 2021).

**Figure 2:**
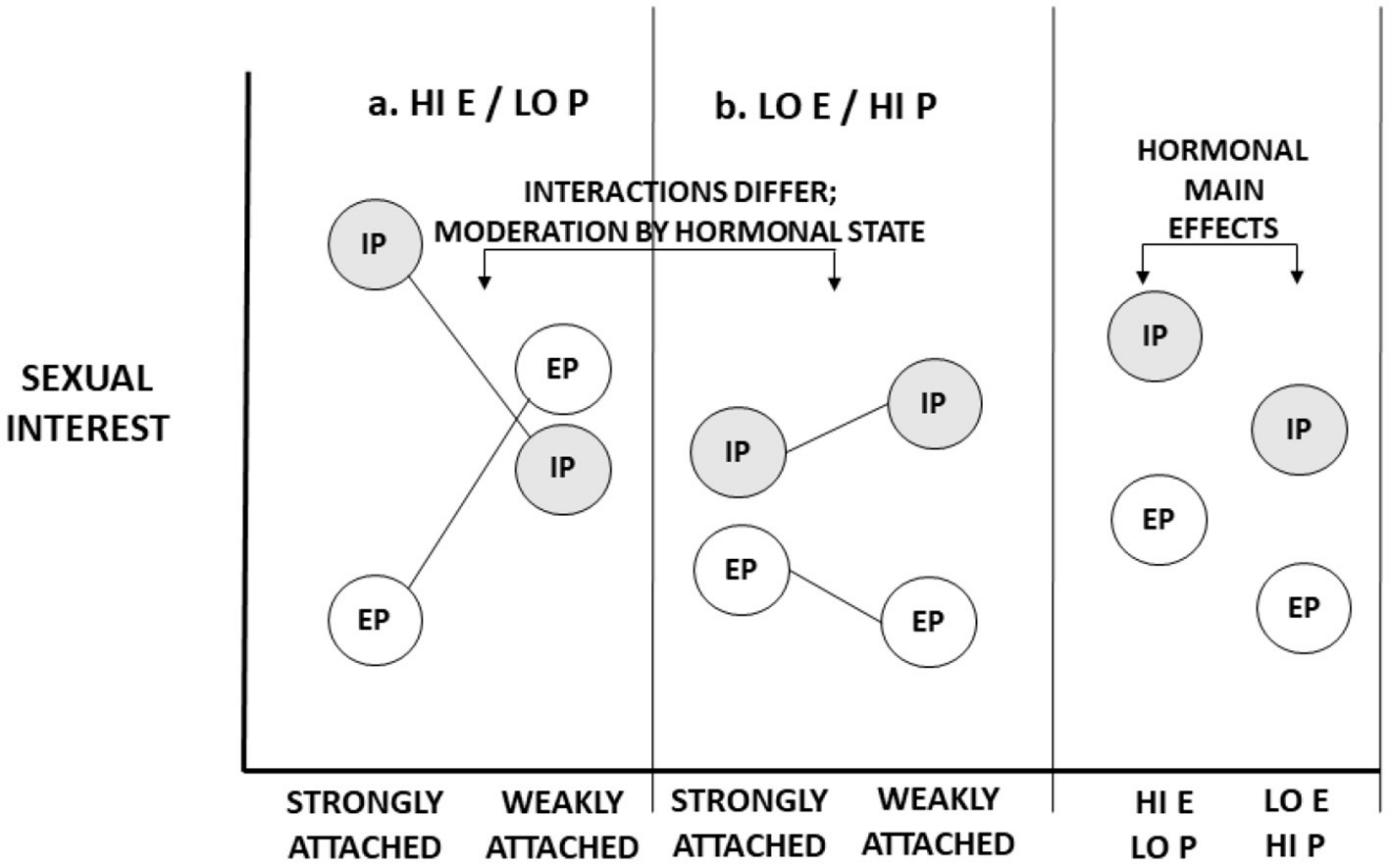
Illustrative representation of the impact of ovarian hormones on the circumstances that evoke sexual interests. Two hormonal states are represented: **(a)** High levels of estrogen (E) and/or low levels of progesterone (P), characteristic of the conceptive, peri-ovulatory phase; **(b)** (relatively) low levels of estrogen and high levels of progesterone, characteristic of the non-conceptive, luteal phase of the cycle. Hormonal states moderate the impact of condition (strength of attachment to partners) on in-pair (IP) and extra-pair (EP) sexual interests. The marginal means of hormonal states for both IP and EP sexual interests are not equal—there is an overall main effect of hormonal state in each—but these main effects are qualified by interaction effects.

Now we consider the possibility that hormonal states affect the circumstances and features that evoke sexual interest. The pattern we illustrate is hypothetical (though see Eastwick, 2009). In Figure 2A, panel A, we illustrate one hypothetical pattern for women with high estradiol and low progesterone levels (characteristic of the conceptive periovulatory phase). Here, when women are strongly attached to partners, women are especially sexually interested in partners. When women are not strongly attached to partners, women have no clear preference for partners. In panel B, we illustrate a pattern for women with low estradiol and high progesterone levels (characteristic of the non-conceptive luteal phase). Here, women strongly attached to partners exhibit relatively little interest in partners, but women weakly attached to partners exhibit somewhat more interest. Hormonal state influences the effects of circumstances and male features on sexual interests. In other words, hormones moderate the conditions that elicit sexual interest.

### Hormonal Influences on “General” Sexual Desire

Roney and Simmons (2013) and Jones et al. (2018a) asked women to report their “general” sexual desire (sexual desire independent of any target specified). Both found that, on average, women reported greater general sexual desire when estradiol levels were higher and/or progesterone levels were lower within women's cycles—i.e., when hormone levels were characteristic of the peri-ovulatory phase. Arslan et al. (2018) similarly found increases in both in-pair and extra-pair sexual interests when women were conceptive. These main effects have been interpreted as strong, consistent evidence for an increase in “general” sexual desire mid-cycle and, hence, evidence for the view that ovarian hormones affect libido.

In fact, these main effects do not constitute compelling evidence that hormones have effects on libido or “general” sexual desire. Consider Figures 1, 2. In each one, overall (marginal) main effects of hormonal state are represented. In Figure 1, which represents hormonal effects on libido, main effects naturally emerge: The main effects of hormones on in-pair and extra-pair sexual interests are identical for both strongly attached and weakly attached women. In Figure 2, which represents hormonal effects on what evokes sexual interests, there also exist main effects of hormones—indeed, *main effects of similar magnitude*. What discriminates these figures are not hormonal main effects. Rather, whether there are hormonal *interaction effects discriminates the two*. To rule out the possibility of hormonal moderation, we must go beyond main effects and examine whether hormones impact how strongly certain conditions elicit sexual interests.

We offer a parallel example. On average across days, traffic in large metropolitan areas tends to move toward city centers in the morning, and toward suburbs in the afternoons. One explanation is that people have a natural inclination to be in the city during the day, an explanation perfectly consistent with this main effect on direction of traffic flow. We know, however, that there is another explanation. Many people work day shifts. And more people work in the city and live in the suburbs that vice versa. There is no general inclination for people to prefer the city during the day. It is simply a matter of when and in what direction people go to and leave work. The empirical patterns that discriminate these two explanations are not main effects; they are interactions. For people who do work night shifts, the opposite pattern likely holds, inconsistent with the “natural inclination” explanation. The same could be true of sexual interests. Although there exists an overall small mean increase in sexual activity mid-cycle, women in certain circumstances and in the presence of particular targets could have more sexual interest on non-conceptive days.

We now turn to discuss the evolution of non-conceptive sex. We argue that, because non-conceptive sex evolved to serve functions distinct from key functions served by conceptive sex, non-conceptive sexual interests likely evolved to be sensitive to conditions other than the primary conditions that elicit sexual interests when women are conceptive.

## Evolutionary Perspectives on the Evolution of Extended Sexuality

Evolutionary psychology uses theories of selection and phylogenetic considerations to guide hypotheses on proximate psychological processes. Such theories can be used to establish plausibility constraints on psychological theories. In light of an understanding of what ancestral selection would have favored and disfavored, some forms of psychological process are unlikely to have evolved and, hence, are implausible (naturally, some unlikely processes *could* exist. However, their existence should lead us to question our understanding of what ancestral selection would have favored, a point to which we later return). As noted earlier, Symons (1979) applied selectionist reasoning to argue that female sexual attraction and desire are unlikely to be indiscriminating.

Using similar applications of selectionist reasoning, we argue that the conditions that evoke women's sexual interests during the conceptive, peri-ovulatory phase may not exactly match the conditions that evoke sexual interests during non-conceptive (e.g., luteal) phases.

### Extended Sexuality: A Phylogenetic Perspective

Sexual receptivity and proceptivity outside of the conceptive phase is referred to as extended sexuality (Rodriguez-Girones and Enquist, 2001)^5^. It is “extended” in two related ways. First, sex during the conceptive phase is a given (even if contingent). Non-conceptive sex, which “extends” outside of the conceptive phase, is found in some species but not others. Second, from a phylogenetic perspective, a state of conceptive *and* non-conceptive sexuality evolved from a prior state of solely conceptive sex (at some point in the lineage, whether evolutionarily distant or recent). Non-conceptive sex evolved as an “extension” of conceptive sex (though influences on extended and conceptive sexuality may differ; see below).

### Extended Sexuality in Non-human Primates

#### “Loss of Estrus”

When Symons (1979) referred to women's “loss of estrus,” he effectively referred to the evolution of extended sexuality in human females. At some point in the lineage leading to humans, females possessed classic estrus and were sexually active only when conceptive (like most mammalian species). More recently, females evolved to be sexually active during both conceptive and non-conceptive phases. The evolution of extended sexuality constituted a loss of (classically defined) estrus.

#### Dixson's Critique

Symons (1979) and others (e.g., Alexander and Noonan, 1979; Benshoof and Thornhill, 1979; Burley, 1979; Spuhler, 1979) generally assumed that, within the human lineage, “loss of estrus” occurred recently in the lineage leading to humans, replaced by “continuous ‘copulability.”' That is, these scholars implied that extended sexuality evolved recently in the human lineage. Indeed, they viewed extended sexuality and the resulting outcome of concealed ovulation as distinctly human qualities, having resulted from selection pressures that were distinct to humans.

Alan Dixson (2009, 2012), probably the world's foremost expert on the comparative study of the reproductive biology of primates, claims that the loss of estrus evolved distantly in the human lineage. Prosimian primates exhibit classic estrus, similar to most mammals. By contrast, the vast majority of monkeys and apes possess some degree of extended sexuality; females are sexually active during some non-conceptive parts of their cycles. On this basis, Dixson (2009) argues that loss of estrus evolved approximately 50 million years ago in the lineage leading to humans in an early simian primate, an event shared with lineages leading to virtually all simian primates (even if, in some lineages, extended sexuality has been largely lost; e.g., female gorillas, though sexually active when pregnant, rarely engage in sex during non-conceptive phases of their cycles; Czekala and Sicotte, 2000). Therefore, loss of estrus can tell us little about selection pressures that *distinctly* shaped human reproductive biology.

#### The Function of Extended Sexuality in Non-human Primates

Extended sexuality in non-human primates can potentially reveal selection pressures that shaped primate reproductive biology. What benefits led to its evolution?

In his massive compendium on comparative primate biology, Dixson (2012) identifies multiple possible functions, depending on the species. In some species, including in the most studied exemplars of Old World and New World monkeys (rhesus macaques and common marmosets), patterns of female proceptivity and receptivity change markedly across sexually active phases: Compared to conceptive phases, females rarely initiate sex during non-conceptive periods; extended sexual activity largely involves female willingness to copulate with males that initiate sex. Potentially, females in some species gain little from extended sexuality other than cost savings. Within these polygynandrous groups, females may pay fewer costs by engaging in undesired, non-conceptive sex than by repeatedly resisting ardent males.

In other species, however, females initiate sex outside of the conceptive phase. Some researchers argue that females in some of these species gain benefits of paternity confusion (Hrdy, 1979; Van Schaik et al., 2000). Males who have not copulated with a female prior to birth of her offspring can rule out their own paternity. Males may hence benefit from harming, even killing, offspring. By mating with all adult males in the group during each cycle, females may mitigate male-instigated harm to offspring. However, multiple mating potentially compromises female choice. Absent cryptic bias of sireship or male competition for sexual access, females risk drawing a sire at random. Females may have evolved to concentrate efforts to confuse paternity through multiple mating during extended sexuality, while exerting adaptive preferences for sire choice when conceptive, either for genetic quality (e.g., Nunn, 1999) or paternal care and protection (e.g., Alberts and Fitzpatrick, 2012). (For possible examples of this function of primate extended sexuality, see Heistermann et al., 2001 [Hanuman Langurs]; Lu et al., 2012 [Phayre's leaf monkeys]; Knott et al., 2010 [orangutans]; Tiddi et al., 2015 [black capuchins; also Dixson, 2012]; Matsumoto-Oda, 1999; Stumpf and Boesch, 2005; Pieta, 2008 [chimpanzees; but see alternative view in Muller et al., 2011])^6^.

How widespread primate extended sexuality for paternity confusion is remains unknown, but exceptions exist. Female Assamese macaques engage in highly extensive extended sexual activity, both during non-conceptive phases of the cycle (characterized by rates of copulation no different from those of conceptive phases) and post-fertilization (Fürtbauer et al., 2011a). Although females are promiscuous and mate with all adult males in a group during a conceptive cycle, males and females frequently form long-term consortships, which account for a substantial portion of all copulations. Males appear unable to detect female conceptive status with certainty, as dominant males do not monopolize conceptive matings; rather, males engage in long-term consortships even during extended sexuality. Synchronous female mating further prevents dominant males from monopolizing matings (Fürtbauer et al., 2011b). Accordingly, male reproductive skew is relatively low (Sukmak et al., 2014). Based on past sexual relations, males infer paternity much better than chance levels and engage in extensive care (largely protection from harassment by other males) of offspring they have sired. This may function as true paternal care, as opposed to mating effort to achieve paternity of future offspring (a function more typical of non-human primates; Minge et al., 2016). Extended sexuality and related adaptations (e.g., suppression of cues of estrus) in this species may have evolved to intensify rates of copulation in consortships, which then translate into direct benefits to females in the form of paternal care (Ostner et al., 2013).

### The Concept of Dual Sexuality

Generally, the conditions that evoke female proceptivity and receptivity during conceptive phases do not perfectly match the conditions that evoke their sexual interests during extended sexuality. This makes sense from a strategy-centered, selectionist perspective. Conceptive sexual interests have been shaped, in part, by selection on outcomes due to the fact that it *is* conceptive. Sex at that time can result in an offspring and, hence, sexual interests have consequences for both fertility scheduling and sire choice. *Conceptive sexuality should embody fitness-enhancing strategies pertaining to regulation of fertility and sire choice*. Non-conceptive sex does not have the same immediate consequences (even if it could have direct benefits and implications for sire choice through pair-bonding). In species with extended sexuality, female proceptivity and receptivity during non-conceptive phases has probably evolved because of functions and benefits other than immediate reproduction. Therefore, *non-conceptive sexuality should embody fitness-enhancing strategies for extracting those benefits (while limiting costs of sex, including the costs of poorly chosen conception)*. Non-conceptive interests could be shaped to look very different or fairly similar to conceptive sexual interests, depending on the nature of benefits of each. But, a priori, these sexual interests are unlikely to be shaped to take *identical* forms.

To capture the idea that conceptive and non-conceptive sexual interests are unlikely to take identical forms, Thornhill and Gangestad (2008) introduced the term, *dual sexuality*. Dual sexuality exists when the psychology underlying conceptive and non-conceptive sexual interests lead them to be differentially evoked by circumstances—i.e., as illustrated in Figure 2. Dual sexuality need not imply completely distinct psychologies; some stimuli that evoke conceptive phase interests may also evoke non-conceptive phase interests. As conception probability is graded, dual sexuality may produce changes across the cycle that are similarly graded; psychological shifts are likely influenced by changing hormone levels. In this perspective, black capuchins, Phayre's leaf-eating monkeys, and, likely, Assamese macaques possess dual sexualities, albeit in non-identical ways.

#### Estrus With Extended Sexuality

Classic estrus, again, exists when females are sexually active only during a restricted period during which they are conceptive. Thornhill and Gangestad (2008) used the term “estrus” to refer to adaptations that characterize female conceptive sexual psychology, even when females also exhibit extended sexuality, to underscore that (a) phylogenetically, extended sexuality evolved as an “add-on” to this conceptive sexual phase, (b) extended sexual interests are likely shaped to be non-identical to conceptive sexual interests, and (c) conceptive sexual interests are likely maintained in species with extended sexuality by some of the same endocrinological mechanisms as in species lacking extended sexuality (i.e., physiological homology exists). In this way, they argued that estrus was not “lost” in simian primates, including humans, despite the evolution of extended sexuality^7^.

Additionally, Thornhill and Gangestad argued that human estrus retained not only physiological homology but also conserved function: sire choice (Thornhill and Gangestad, 2008). Upon reflection, we think that they had a useful, valid point but overstated it. While estrous adaptations importantly function to direct sire choice even in species with classic estrus, sire choice can be traded-off against other potential benefits, such as opportunity costs incurred by mate search. Estrous adaptations should function to manage those trade-offs too. As well, estrous sexuality should regulate fertility. Even if sex is potentially conceptive and a suitable partner is available, timing of reproduction may not be opportune (e.g., Dinh et al., 2017, 2021b). Furthermore, species that evolved extended sexuality are not a random set of species. In these species, female sexuality has benefits other than conception. Although those benefits led to the evolution of extended sexuality, they may (depending on the species) also be garnered through conceptive sex, which can lead to adaptive modifications in conceptive sexuality. For example, in Assamese macaques, both non-conceptive and conceptive sex can cement consortships that pay off in currencies of paternal care (contingent on sireship). Adaptations shaped by these benefits may come to be part of estrous sexuality, even if sire choice and fertility regulation adaptations are retained (for an argument along these lines for humans, see Eastwick, 2009; Eastwick and Finkel, 2012).

## Women's Extended Sexuality

While most simian primate females are sexually active outside of the conceptive phase, women are extreme exemplars, being sexually active throughout the entire cycle. Evolution-minded thinkers consider the evolution of human extended sexuality a potential key to understanding the forces of selection that forged our mating patterns, patterns of parental investment, and our social nature.

### The Focus on Concealed Ovulation

Early on, Symons, Alexander, and other scholars who portrayed women's extreme extended sexuality in high relief focused on one particular effect—the “concealment” of ovulation. This focus was, in hindsight, both insightful and misleading. Consistent with selection for an “undisclosed” conceptive status^8^, women appear to be designed to minimize generalized male access to cues of their conception risk. Yet there could be “leaky cues”—weak cues that are likely not designed to advertise conceptive status to others (Thornhill and Gangestad, 2008; Haselton and Gildersleeve, 2011). However, “concealed ovulation” does not imply that women are incapable of (unconsciously) discriminating their cycle phase. Women likely have mechanisms to discern and functionally respond to changes in conception probability. For instance, even though women are sexually active across the cycle, their sexual psychology and experiences (e.g., the nature of their sexual interests) change.

Furthermore, the idea that women “conceal” ovulation suggests that other female primates fully advertise it, which is not true. Sexual rump swellings have independently evolved at least three times in Old World primates (Pagel and Meade, 2006). Females in these species also exhibit extended sexuality. Sexual swellings arise prior to when females are conceptive in their cycle and persist past ovulation. Although swellings tend to be maximal during the peri-ovulatory phase, the association is far from perfect. Sexual swellings are “graded” signals that, *by design*, are highly imperfect indicators of conception status (Nunn, 1999). Female primates with extended sexuality benefit from it only when males lack access to perfect cues of conceptive status (i.e., female conceptive status is not fully detectable).

Many, if not most, females that display sexual swellings also potentially benefit from extended sexuality through paternity confusion. In some species that did not evolve sexual swellings, females may exhibit other signals. New World female monkeys that may confuse paternity through extended sexuality (e.g., black capuchins) exhibit behavioral displays (e.g., ritualized vocalizations) that may similarly function as imperfect, graded signals (Tiddi et al., 2015). In both Old World and New World species of this sort, females may benefit from attracting maximal male sexual interest during times surrounding ovulation. This way, females can actively or passively (as a result of outcomes of male intrasexual competition) select desired sires when conceptive, while confusing paternity when non-conceptive.

### The Evolution of Women's Extended Sexuality

Comparatively, the fact that women lack sexual swellings or ritualized behavioral cues overlapping with the conceptive phase is revealing about the potential function of their extended sexuality. It likely has not been shaped to facilitate paternity confusion. Strassmann's (1981) explanation of concealed ovulation, broadened to include extended sexuality, may offer one plausible scenario for the evolution of women's extended sexuality. Strassmann argued that, in a species in which males may potentially engage in true paternal care, males most likely to benefit from caring are non-dominant males. Following successful reproduction, males can choose to invest in care or re-enter the mating market to compete for another fertilization (or some combination of these two). The benefits of engaging in care partly depend on the net benefits (e.g., the rate of success) of competing for mating (e.g., Kokko and Jennions, 2008). Non-dominant males benefit less from competing and, therefore, are most likely to benefit from investment in care. As a result, females evolved to prefer non-dominant males as sires for the benefits of paternal care. Accordingly, concealing their conceptive status prevented dominant males from systematically monopolizing conceptive matings through intrasexual competition against non-dominant males (for a discussion of potential contributions of female-female intrasexual competition to the evolution of undisclosed conceptive status in humans, see Krems et al., 2021).

As Kokko and Jennions (2008) emphasize, male benefits to investment in care also crucially depend on paternity assurance. In Strassmann's scenario, the concealment of ovulation permits non-dominant males to become sires but also limits *their* access to cues to conceptive status. Hence, males do not attain paternity assurance through detection of fertility cues. Rather, paternity assurance is achieved through regular sexual access throughout the cycle of a target female (some portion of which is conceptive), in conjunction with reasonable confidence that other males have not engaged in sex with that female during the cycle^9^.

According to this perspective, women's extended sexuality has been shaped to assure paternity of a primary mate to promote male investment in offspring. This view converges with a more general framework for the evolution of pair-bonding and biparental care in humans, supported by modeling (Gavrilets, 2012). In this framework, the evolution of paternal care in humans was led by non-dominant males, who could benefit most from care. Females preferentially mated with such males to obtain care. Ultimately, most dominant males also evolved to care for offspring to attain paternity, given female preferences for males who will invest in offspring (a portion of dominant males will still benefit from substantial mating effort and successful multiple mating outside of pair-bonds; see Gavrilets, 2012, for details).

Regardless of the specifics involved in the evolution of women's extended sexuality and whether these models are correct, we emphasize that extended sexuality evolved for functions other than direct conception. Strassman's explanation offers one possible function of women's extended sexuality, though it has yet to be fully evaluated. (Even if it possesses some truth, it may not fully explain extended sexuality. E.g., unmated women possess extended sexuality too; what functions does extended sexuality serve them? And, for an alternative view that extended sexuality draws male attention and possibly sperm away from other females in polygynous relationships, see Geary et al., 2011). Importantly, because women's extended sexuality evolved for reasons other than the implications of sex for immediate reproduction, the circumstances that evoke sexual interest during extended sexuality are unlikely to be precisely the ones that evoke sexual interest during estrus.

## Evaluations of Women's Dual Sexuality

We have argued that, based on fundamental conceptualizations regarding functional (and hence strategic) design, women likely possess a dual sexuality. But existing evidence is not yet definitive for answering whether women have dual sexuality. Additional empirical—and, importantly, conceptual—work is needed. Below, we briefly discuss pertinent empirical findings and ideas.

### Genetic Benefits for Offspring

The dual sexuality framework is a broad umbrella, with specific theory to be filled in. Generally, conceptive sexuality should be concerned with fertility regulation (timing of reproduction) and sire choice (choosing a right male as a father). One prominent theory along these lines asserts that, when women are conceptive, they have increased preference for male features that may have ancestrally been indicators of genetic quality—indirect benefits that sires pass to offspring. Researchers have examined preferences for candidate features of genetic quality, some of which are directly involved in regulating male-male competition: the body scents of symmetrical men (Gangestad and Thornhill, 1998; Rikowski and Grammer, 1999; Thornhill and Gangestad, 1999; Thornhill et al., 2003); facial masculinity (e.g., Penton-Voak et al., 1999); vocal masculinity (Puts, 2005; Feinberg et al., 2006; Pisanski et al., 2014); facial symmetry (though findings were largely negative; e.g., Koehler et al., 2002); body masculinity (e.g., Little et al., 2007); facial features associated with testosterone (Roney et al., 2011); behavioral indicators associated with dominance, confidence, and “social presence” (Gangestad et al., 2004, 2007; Flowe et al., 2012; Giebel et al., 2013; Cantú et al., 2014).

A meta-analysis and *p*-curve analysis suggested that non-zero true effects in targeted preferences exist (Gildersleeve et al., 2014a,b; cf. Wood et al., 2014). At the same time, recent large-scale replication studies and studies examining hormonal influences have not been encouraging (e.g., Dixson et al., 2018; Jones et al., 2018c,d; Jünger et al., 2018a,b; Marcinkowska et al., 2018; Stern et al., 2019, 2020, 2021). Most effects appear to be in predicted directions. Some have been suggestive of true effects (e.g., on estradiol-vocal masculinity preference links: Jones et al., 2018c; on progesterone-body muscularity links: see Dinh et al., 2021a, on Stern et al.'s, 2021, study). However, effects have typically been small and non-significant. Small true effects cannot be ruled out. (We think small true effects are consistent with the overall findings. Conversely, an across-the-board null hypothesis does not explain the *p*-curve results or why predicted effects are sometimes significant, whereas significant effects in the direction opposite that predicted have not been reported). Large, robust effects that early work suggested are not consistent with findings. We return later to briefly comment on these findings.

### Pair-Bonding and Estrous Sexual Psychology

The dual sexuality framework is broad and encompass multiple distinct theories. Even if one theory is wrong, others may be right. Above, we described how females often benefit from choosing non-dominant males as sires because these males are more likely to care for offspring, which may have led to the evolution of human extended sexuality. Undisclosed conceptive status prevents dominant males from monopolizing conceptive matings, permitting females to choose non-dominant males willing to invest in care as sires (Strassmann, 1981). At first blush, these statements may appear to be incompatible with the idea that women retain estrous preferences for dominant and intrasexually competitive males.

However, women's preferences for sire choice may be conditional. Women may act on estrous preferences for dominant males very selectively and only under certain conditions. Perhaps most of the time, a woman in a valuable relationship with a highly-investing partner would have benefitted most from conceiving with her partner. Thornhill and Gangestad (2008) made this point:

We have characterized estrous sexuality in terms of adaptations designed to garner good genes for offspring. Much evidence speaks to the existence of those adaptations in women (see Chapters 9 and 10). … [More broadly, however], estrous sexuality should generally function to enhance adaptive sire choice by females. One component of adaptive sire choice is choice of a partner who can deliver genetic benefits to offspring. But *in pair-bonded species, in many instances the best sire for a woman's offspring is in fact the pair-bond mate*, and not merely in instances in which the mate has good genes; the primary partner delivers non-genetic material benefits in a variety of currencies (Chapter 4) and loss of those benefits could have a drastic negative impact on a female's fitness. [pp. 307-308; emphasis added]

A year after, Eastwick (2009) proposed his adaptive workaround hypothesis. Because of the value of strongly bonded relationships, he conjectured, women evolved adaptations that function to strengthen highly valued relationships when conceptive in their cycles. Eastwick and Finkel (2012) found that women in strong pair-bonds experienced more intimate sex with partners when conceptive and were less likely to say they would act on extra-pair attraction. Eastwick proposed that the function of these changes is to solidify pair-bonds, as a counterforce to estrous adaptations that might weaken bonds. Alternatively, results could also be consistent with Thornhill and Gangestad's (2008) claim that women may have evolved to select highly investing partners as sires.

Recently, we and some colleagues sought to assess whether partnered women's bond strength (or, as we refer to it, “loving attachment” to partners) moderates the impact of ovarian hormones on their in-pair and/or extra-pair sexual interests. We assessed urinary estrogen and progesterone levels in 181 women who participated in up to 4 sessions, across a period of about a month. In an initial session, women completed extensive questionnaires assessing their relationship qualities (both from their perspective and the perspective of their partners). Loving attachment strongly interacted with within-woman progesterone levels to predict in-pair versus extra-pair sexual interests. Simple main effect analyses showed that, when women were strongly attached to partners (1*s* above the mean), low progesterone levels (characteristic of the follicular and peri-ovulatory phases) were associated with low levels of extra-pair interests relative to in-pair sexual interests. Furthermore, low progesterone levels were associated with greater rates of initiation of sex with partners for these women. For women relatively unattached to partners (1*s* below the mean), a contrasting pattern held: When progesterone levels were low, their extra-pair interests tended to be greater compared to when their progesterone levels were high (Dinh et al., 2022b), potentially consistent with the view that, for these women, mid-cycle increases in extra-pair attraction partly reflect interest in indicators of genetic quality (e.g., Durante et al., 2016).

These empirical patterns illustrate the same point made by Figure 2: an ovarian hormone moderates the impact of a circumstance—the level of relationship attachment—on in-pair versus extra-pair sexual interests. It does not fit the pattern in Figure 1.

### Relationship Features and Extended Sexuality

To offer predictions about changes in women's sexual interests across the cycle, we must have some understanding of both estrus and extended sexuality. Women's extended sexuality should have been shaped to enhance the benefits that led to its evolution.

Based on the notion that women's extended sexuality has been selected to foster investment by primary partners, Grebe et al. (2013) argued that women's sexual interests during non-conceptive phases should be particularly sensitive to relationship features. Specifically, Grebe et al. (2013) suggested that women should be particularly interested in bolstering investment from primary partners—and hence initiate sex with partners more during extended sexuality—when women are highly invested in their relationships but their partners' investment lags behind their own. A within-woman study of very modest sample size (50) yielded support for these ideas (see also Sheldon et al., 2006; Grøntvedt et al., 2017).

In the study of 181 women mentioned above, we conducted analyses that further tested these notions. We did not find support for the prediction that women's overall relationship investment, relative to their partners' investment, predicts in-pair versus extra-pair sexual interests when hormone levels are characteristic of the non-conceptive luteal phase (high progesterone levels relative to estrogen levels). In preregistered exploratory analyses, however, we found that estrogen levels moderated the impact of discrepancies in women's versus their partner's levels of romantic passion. When women were more passionate about their partners than their partners were about them, women experienced greater in-pair sexual interests, relative to extra-pair interests, when their estradiol levels were relatively low—i.e., characteristic of extended sexuality (Dinh et al., 2022b).

The function of romantic passion is purportedly distinct from that of loving attachment. Strong pair-bonding—a state of interdependence in which both partners regard the interests of their partners to be their own interests—purportedly facilitates efficient biparental care of offspring (e.g., Fletcher et al., 2015). Romantic passion (also referred to as romantic love, passionate love, or limerence; Tennov, 1999) functions in the context of pair-bond formation. It promotes pair-bonding through motivational impacts and/or signaling-of-commitment (e.g., Fisher, 1998; Galperin and Haselton, 2010). Romantic passion may ultimately lead to loving attachment, after which romantic passion may wane, its function fulfilled. When women express greater levels of romantic passion than their partners, they signal that they are more interested in developing a strong pair-bond than their partners are. If extended sexuality evolved in part to foster male interest in greater investment, female-male discrepancies in levels of romantic passion plausibly predict women's extended sexual interest in partners, relative to other men.

### The Impact of Male Attractiveness

Although hormonal moderation of the impact of various relationship qualities on in-pair versus extra-pair sexual interests has not received a lot of attention to date, one moderation effect has received a good deal of attention. As might be expected, male partners' sexual attractiveness negatively associates with women's interests in extra-pair men; women with “sexy” partners express less interest in other men, compared to women with relatively “unsexy” partners. Multiple studies have found that this effect is stronger when women are conceptive in their cycles (e.g., Pillsworth and Haselton, 2006; Larson et al., 2012; Dinh et al., 2022a; see Gangestad and Dinh, 2021, for a review). The purported explanation is that women place greater value on sexually attractive male features when conceptive in their cycles, presumably because these features were associated with genetic benefits to offspring ancestrally. Recently, Arslan et al. (2018) claimed to find no support for this moderation effect. In fact, however, two coding errors distorted results. When these coding errors were corrected, analyses on Arslan et al.'s sample using their most powerful method of assessing conception risk (a continuous measure; Gangestad et al., 2016) yielded considerable support for this moderation effect (Gangestad and Dinh, 2021; but see also response by Arslan et al., 2021).

In our recently collected dataset of 181 women, we examined moderating impacts of ovarian hormone levels on the association between male partners' attractiveness and women's extra-pair sexual interests (Dinh et al., 2022a). Analyses found that when women's progesterone levels were low—characteristic of the follicular and peri-ovulatory phase—partner attractiveness more strongly predicted extra-pair interest in a negative direction, consistent with previous effects. However, some preregistered effects examined were near-zero. All in all, the null hypothesis that there exist no effects is a very poor explanation of the overall pattern of results, even if we currently do not fully understand the reasons for variable effects (Dinh et al., 2022a,b)^10^.

This moderation effect, if robust, begs a question. This effect assumes that women differentially value sexually attractive features across conceptive versus non-conceptive phases. Yet, as we discussed above, large-scale replication studies have not found strong, compelling evidence for hormonal moderation of mate preferences (e.g., for muscular bodies). Durante et al. (2016) suggested one possible resolution. Perhaps mate preference shifts are moderated. For instance, women who are strongly attached to partners may express relatively little interest in extra-pair men when conceptive. It may make sense that these women also show little evidence of increased interest in, say, muscular men when conceptive. The same may apply for women with attractive partners—or, more generally, women who show relatively little interest in extra-pair men during conceptive phases for other reasons. By contrast, when women do express interest in extra-pair men when conceptive, they may be particularly interested in muscular men. This may be one reason why preference shifts are weak overall and not consistently detected. It also remains untested to date.

### Clarifications

Based on a priori ideas about how selection has shaped women's estrous sexuality and extended sexuality, we have argued that it is unlikely that women's conceptive and non-conceptive sexual interests are evoked by precisely the same conditions. This possibility has not been extensively explored. We have briefly discussed evidence arising from recent empirical work, guided by general notions about dual sexuality, that are consistent with hormonal moderation effects on the conditions that evoke women's in-pair and/or extra-pair sexual interests.

We emphasize two points. First, the findings we discuss are provisional. We have not discussed findings in detail, as the papers that present these results must still undergo peer review and evaluation. Furthermore, some findings arose from analyses that were not preregistered or were preregistered as exploratory. Future research may further evaluate the robustness of these findings.

Second, we present these results as illustrative of findings consistent with dual sexuality. The framework is compatible with a number of distinct theories about the contrasting functions of estrus and extended sexuality. We encourage further development of selection-based theories that propose differences in what conditions elicit sexual interests during estrus versus extended sexuality.

To illustrate different predictions made by the motivational priorities perspective and certain theoretical variations that fall under the dual sexuality framework, we include Table 1. It focuses specifically on predictions made about moderating effects of relationship qualities (see the above sections, “Pair-bonding and estrous sexual psychology” and “Relationship features and extended sexuality”).

**Table 1:** Illustrative effects that distinguish the motivational priorities perspective and the dual sexuality framework.

**Perspective**	**Expectations regarding hormonal interactions with** **condition**
Motivational priorities	Few, if any, hormonal interactions on sexual desires are expected. For women in romantic relationships, estradiol should be positively associated with and/or progesterone levels should be negatively associated with sexual interests in both in-pair and extra-pair partners. Conditions (such as loving attachment) may have independent effects on in-pair and extra-pair sexual interest, but these effects should not generally be moderated by estradiol and/or progesterone levels.
Dual sexuality	Some hormonal interactions with condition should exist, though different views of estrous and extended sexuality offer different predictions about the nature of these interactions. *Sire choice and fertility regulation favoring partners women are* *strongly attached to*. When women are strongly attached to romantic partners, women's sexual interests should be especially focused on in-pair partners, relative to extra-pair partners, when estradiol levels are high and/or progesterone levels are low (i.e., during estrous sexuality). See Dinh et al. (2022b). *Pair-bond extended sexuality theory*. When women are more involved in their relationships than partners are, women's sexual interests should be especially focused on in-pair partners relative to extra-pair partners, when estradiol levels low and/or progesterone levels are high (i.e., during extended sexuality). See Dinh et al. (2022b).

## What if the Conditions that Evoke Conceptive and Non-conceptive Sexual Interests Do Not Differ?

Multiple lines of research suggest that women's estrus and extended sexuality are distinct sexualities. However, existing evidence has not convincingly demonstrated through repeated replication that distinct conditions differentially evoke conceptive and non-conceptive sexual interests. What if it turns out there are no differences between the conditions that evoke conceptive and non-conceptive sexual interests?

Such a state of affairs should have clear consequences for theory, as it should lead us to question the a priori basis for expecting differences. *A priori*, if the functions of conceptive and non-conceptive sexual interests differ, conceptive and non-conceptive sexualities were shaped by selection to strategically garner the benefits (and limit the costs) of each; this implies that conceptive and non-conceptive sexual interests should be elicited by different circumstances.

Are there scenarios in which these expectations would be wrong? We are not sure. If these expectations are wrong, evolution-minded scholars should attempt to explicate the selection pressures that would have shaped women's sexual interests to yield the empirical patterns observed. Perhaps alternative understandings of what selection would have favored are needed. But for theoretical progress to be made, those alternatives must be spelled out.

## Summary Remarks

The current literature on shifts in women's sexual interests across the cycle is rife with conflicting findings. No one theoretical perspective is widely accepted. We have discussed what is known and what is not known and reflected on key issues.

First, despite conflicting findings, one general empirical pattern appears to be firmly established: On average, women experience greater sexual desire and interest when conceptive in their cycles than when non-conceptive. Hormonal changes likely are a proximate process accounting for these shifts (Roney and Simmons, 2013).

Second, the ultimate reasons underlying these empirical patterns on changes in sexual interests are not straightforward or self-explanatory. One perspective views hormonal signals as directly influencing “general” sexual desire, or libido. We argue that, alternatively, hormones potentiate or de-potentiate the effect of conditions on target-specific sexual interests. The existence of main-effect associations between hormone levels and sexual desires are compatible with both perspectives. The perspectives differ with respect to whether they expect distinct conditions to differentially evoke sexual interests as a function of conceptive status.

Third, we make the key point that extended sexuality evolved to serve functions distinct from the functions of estrous sexuality. We discussed the evolution of extended sexuality, provided evidence from non-human primate examples, and addressed possible functions of women's extended sexuality. Different functions demand different strategies to attain benefits. *A priori*, based on evolutionary logic, we argued that the conditions that evoke sexual interest across conceptive and non-conceptive phases likely differ.

Fourth, our understanding of women's sexuality, including possible differences between estrous and extended sexuality, is not well established. We discussed illustrative lines of research that examine hormonal moderation of conditions that affect sexual interests. More work is needed along these lines. Unless potential moderation is explored, we cannot conclude that important moderation effects do not exist.

Patterns of change and stasis in women's psychology across the cycle likely reveal important truths about selection, which has shaped many features distinct to humans. After four decades of rather intense theoretical and empirical scrutiny, consensual understanding of these patterns remain elusive. Tremendous progress has been made, though firm conclusions cannot yet be drawn regarding cyclic shifts in women's psychology and behavior. Much more empirical research and theoretical developments are necessary for a thorough and nuanced understanding of human mating and sexuality.

## Data Availability Statement

The datasets presented in this article are not readily available because the article presents preliminary results. However, this article is primarily a conceptual paper and not an empirical research paper. All preliminary findings will ultimately be reported in papers that will give access to all pertinent data uploaded to a publicly accessible repository. All data will be de-identified. Requests to access the datasets should be directed to Steven Gangestad sgangest@unm.edu.

## Ethics Statement

The studies involving human participants were reviewed and approved by University of New Mexico, Main Campus IRB. The participants provided their written informed consent to participate in this study.

## Author Contributions

Both authors listed have made a substantial, direct, and intellectual contribution to the work and approved it for publication.

## Funding

This work was supported in part by the National Science Foundation, Grant Number 1729856. TD was partially funded by an NSF Graduate Research Fellowship.

## Conflict of Interest

The authors declare that the research was conducted in the absence of any commercial or financial relationships that could be construed as a potential conflict of interest.

## Publisher's Note

All claims expressed in this article are solely those of the authors and do not necessarily represent those of their affiliated organizations, or those of the publisher, the editors and the reviewers. Any product that may be evaluated in this article, or claim that may be made by its manufacturer, is not guaranteed or endorsed by the publisher.

## Footnotes

^1^Most studies have examined subjective sexual desire or measures of psychological interest, which this generalization pertains to. Few have examined genital arousal, which often does not correspond to psychological motivation, especially in women (Chivers et al., 2010). One exception is Suschinsky et al. (2014), who found a marginally significant increase in genital responses to sexual stimuli during the conceptive phase. Moreover, consistent with this general theme, some studies have shown an order effect, such that stimuli shown first during a conceptive phase elicit greater sexual interest subsequently, compared to stimuli first shown during the luteal phase (Slob et al., 1991; Wallen and Rupp, 2010; Suschinsky et al., 2014). We note that empirical exceptions exist. For instance, in a small sample of 33 partnered women, Grebe et al. (2016) reported that progesterone levels positively predict women's sexual interests in their partners. In a study tracking 30 partnered women through 15 consecutive days of a cycle, Righetti et al. (2020) found that estradiol levels negatively predicted women's sexual interests in partners. Though contrary to general trends, these patterns may reflect true effects. Results may reflect moderation effects of relationship qualities not yet fully understood.

^2^A full explication of an incentive perspective on sexual motivation and its implications is not possible here, but we note that (a) this view is a broad one, and does not entail specific ideas about the nature of stimuli that are attractive or the processes that affect attraction (e.g., relatively “hard-wired” vs. learned responses); Toates (2009, 2014) explicitly places this view in an evolutionary psychological framework, with the sexual response system involving modular components, (b) in this view, sexual fantasy can give rise to sexual desire through imagined presence of eliciting circumstances, (c) sexual desire is subject to both excitatory and inhibitory processes, understood within a more general hierarchical control system view (see, e.g., Gallistel, 2013); attraction, then, can be inhibited (Finkel, 2014).

^3^In philosophy of science, a dispositional state is one that, given an additional event, will lead to an outcome (e.g., Carnap, 1937; Goodman, 1954). A glass has the disposition to break when dropped on a hard surface. It may never break—but it has the possibility of breaking under specified conditions. In the same way, internal states referred to here are dispositional with respect to sexual desire: An individual under such a state has the possibility of experiencing sexual desire, but actualization of that possibility requires that specific conditions be met.

^4^As Thornhill and Gangestad (2008) also argued, it is not clear that female sexual attraction is essential to insemination. Males should be strongly selected to seek conceptive females to mate with. Female attraction to a male is often not required for a male-female encounter to occur. Females only need to be receptive to male solicitation. Women, in fact, can experience physiological and reflexive changes associated with receptivity without experiencing psychological sexual desire (Laan and Everaerd, 1995). As female sexual attraction and desire are not necessary for indiscriminant insemination, female sexual attraction and desire probably function to influence sire choice and avoid indiscriminant insemination. We clarify that Roney's (2018, 2021) view is not that sexual interest itself is indiscriminate. It fully recognizes that women have preferences for some potential mates over others. But it argues that the impact of ovarian hormones on sexual desire is *general* and, hence, indiscriminate; estrogens purportedly increase the attractiveness of men in general, whereas progesterone purportedly suppresses their attractiveness in general (e.g., Jünger et al., 2018a).

^5^Sexual receptivity and proceptivity during pregnancy and lactation qualify as extended sexuality (see, e.g., Magganetti and Pillsworth, 2020). Here we focus on sexual receptivity and proceptivity during non-conceptive phases of an ovarian cycle.

^6^Naturally, for extended sexuality to function effectively in these species, males must not be able to discern when females are conceptive with certainty, even if some predictive but fallible markers of female conceptive status exist.

^7^This point of view may seem to contrast with Dixson's (2009, 2012) argument that estrus was lost in an early simian primate. However, the contrast is merely a semantic one. (In Dixson's view, estrus is lost whenever extended sexuality in a cycle evolves – but that of course is not what Thornhill and Gangestad (2008) meant by estrus when claiming it persists).

^8^Thornhill and Gangestad (2008) preferred the term, ‘undisclosed conceptive status,” for several reasons: first, what is important is not that *ovulation* is concealed but, rather, conception risk, which precedes ovulation in the cycle; second, conceptive status is not advertised, yet males generally discern conceptive status via byproducts of other adaptations. Thornhill and Gangestad based this view on modeling by Pagel (1994) showing that costly female signals of conceptive status will not evolve so long as males have access to byproduct cues. Although Thornhill and Gangestad hence argued that sexual swellings likely advertised quality or capacities for successful reproduction, they did permit that females could possibly derive a net benefit from inciting male intrasexual competition through a fallible “fertility signal” (e.g., Nunn, 1999). Based on evidence, we think the latter is likely (though the costliness of sexual swellings remains puzzling, as a cheaper alternative [e.g., the vocalizations of black capuchins] could seemingly derive the same benefit).

^9^As discussed earlier, Assamese macaques exhibit forms of mating that are rare for an Old World monkey. Although females mate promiscuously, males and females form prolonged consortships and copulate throughout female cycles. Unlike most macaques, females evidence little sexual swelling. Fathers may engage in true paternal care, largely in the form of protection from other males. Although paternal care is hardly as extensive as that found in most human societies, its evolution may be supported by similar kinds of sexual adaptations, independently evolved in the lineage: relatively undisclosed conceptive status, extreme extended sexuality, greater paternity assurance to consorts through repeated copulation throughout the cycle (and, indeed, after conception).

^10^Outcome measures that yielded effects tapped *absolute* levels of *intensity* of extra-pair sexual interest. Ones that did not tapped *frequency* of sexual interests *relative* to that of typical days. Perhaps one of these features explain variability in findings, though this possibility remains speculative.
